# High loss factor piezoelectric damping composite with three-dimensional reduced graphene oxide as the conductive phase†

**DOI:** 10.1039/c8ra00175h

**Published:** 2018-04-03

**Authors:** Wenchao Xue, Hua Li, Roberto Dugnani, Hafeez Ur Rehman, Chunmei Zhang, Yujie Chen, Hezhou Liu

**Affiliations:** Key Laboratory of Metal Matrix Composites, School of Materials Science and Engineering, Shanghai Jiao Tong University China; Collaborative Innovation Center for Advanced Ship and Deep-Sea Exploration, Shanghai Jiao Tong University China; University of Michigan, Shanghai Jiao Tong University Joint Institute China

## Abstract

In this study, a lead zirconate titanate (PZT)/*in situ* polymerized polyurethane (PU) composite with three-dimensional (3D) reduced graphene oxide (rGO) as the conductive phase was prepared and the potential of 3D rGO to enhance the damping properties was investigated. The conductivity and damping properties of the composite were systematically investigated. The results show that the conductive threshold of the composite is reached at a very low rGO content of about 0.7 wt% by using the 3D rGO structure. The best damping performance of the piezoelectric damping composite is achieved at the conductive threshold, where the loss factor is 0.22 (almost 41%) higher and the temperature range where tan *δ* ≥ 0.3 is 13.2 °C (almost 84%) wider than those of the PU matrix. A composite consisting of only PU and rGO sheets without the 3D structure was prepared for comparison. The conductive threshold of this composite is more than 0.9 wt% and the highest tensile strength is 5.63 MPa when the rGO content is 0.6 wt%, indicating that the 3D structure reduces the use of the conductive phase and does not significantly affect the tensile strength of the matrix.

## Abstract 2

Piezoelectric damping composite with 3D rGO as the conductive phase that possesses good damping properties with the conductive threshold as low as 0.7 wt%.

## Graphical abstract

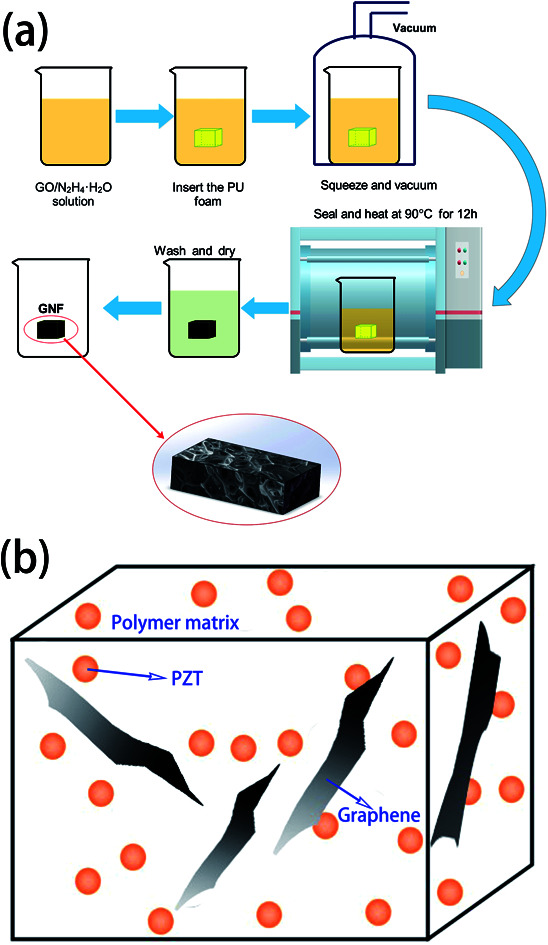

## 1. Introduction

Vibration in modern equipment is often undesirable because of the demand for accuracy, reliability, durability, stability, and noise reduction. Therefore, there is strong interest in novel materials that can lead to efficient vibration reduction. Polymers are generally preferred because their viscoelastic behavior provides higher damping performance than cement, metals, and alloys.^1^ However, the ideal damping performance of polymers can only be obtained in a narrow temperature range of about several tens of degrees near the glass transition temperature. Piezoelectric damping composites have been developed by introducing a dissipation way of force–electricity–heat to meet higher demands. They are very promising for both active and passive vibration control in many industries, such as the automobile, airplane, aerospace, railway, sports, and engineering industries.^2^

The first piezoelectric damping composite with a piezoelectric ceramic/polymer structure was reported by Forward.^3^ According to the theory of piezoelectric shunt damping, adding an appropriate piezoelectric component and a conductive phase to damping materials can be advantageous for energy dissipation.^3–6^ A number of composites with metals,^7^ carbon black (CB),^8–12^ carbon fibers,^13–16^ carbon nanotubes,^17–21^ and graphene as the conductive phase have been prepared and investigated. However, to achieve the conductive percolation threshold, a lot of the conductive phase needs to be added, which may affect the performance of the matrix. For example, Qiao *et al.*^12^ fabricated a piezoelectric damping composite composed of bromobutyl rubber as the matrix, lead zirconate titanate (PZT) as the piezoelectric phase, and acetylene CB (N550) as the conductive agent. However, the conductive percolation threshold of CB is relatively high (almost 8 wt%). Liu *et al.*^22^ prepared chlorobutyl rubber/poly(ethyl acrylate)/piezoelectric ceramic/CB composites with 10 wt% CB content. Shamir *et al.*^23^ used dodecyl benzene sulfonic acid to improve the dispersion of CB in poly(styrene-*rand*-butyl acrylate) copolymers, which decreased the percolation threshold. However, the CB content was still almost 3 wt%. Furthermore, according to the literature, there is a negative correlation between the damping performance of composites and the CB content, especially when the CB content is more than 4 wt%.

Here, to overcome the unfavorable effects of a high conductive phase content, a new damping composite material containing a conductive phase with three-dimensional (3D) connectivity (reduced graphene oxide, rGO) is reported. Various 3D structures can be used as the conductive phase, such as graphene aerogels,^24–26^ polymer/graphene hybrid aerogels,^27^ and polymer-based graphene foams.^28^ Because the 3D conductive structure needs to be composited with polyurethane (PU)/PZT, a matrix with high strength and large voids is required, so PU foam was chosen as the matrix to fabricate 3D rGO. 3D rGO was prepared by a modified Hummers method and hydrothermal reduction. Using these techniques, rGO self-assembled on the PU matrix to form a 3D conductive network. PZT was chosen as the piezoelectric phase because of its excellent piezoelectric properties, high Curie temperature, and high polarization and electromechanical coupling coefficients.^29^ PU was used as the matrix material because of its good damping properties and to adjust the glass transition temperature and loss factor by modifying the ratio of the soft and hard segments.

## 2. Materials and methods

### 2.1 Synthesis of 3D rGO and rGO sheets

Hydrazine hydrate (85 wt%) was purchased from Sinapharm Chemical Reagent Co., Ltd., China. The PU foam was commercial PU sponges supplied by Sichuan Hongchang Plastics Industrial Co., Ltd., China. The graphene oxide (GO) solution was prepared by a modified Hummers method.^30^ The commercial PU sponges were first rinsed in acetone and distilled water for 15 min using an ultrasonic cleaner. The sponges were then dried in an oven at 70 °C for 12 h.

**Fig. 1:**
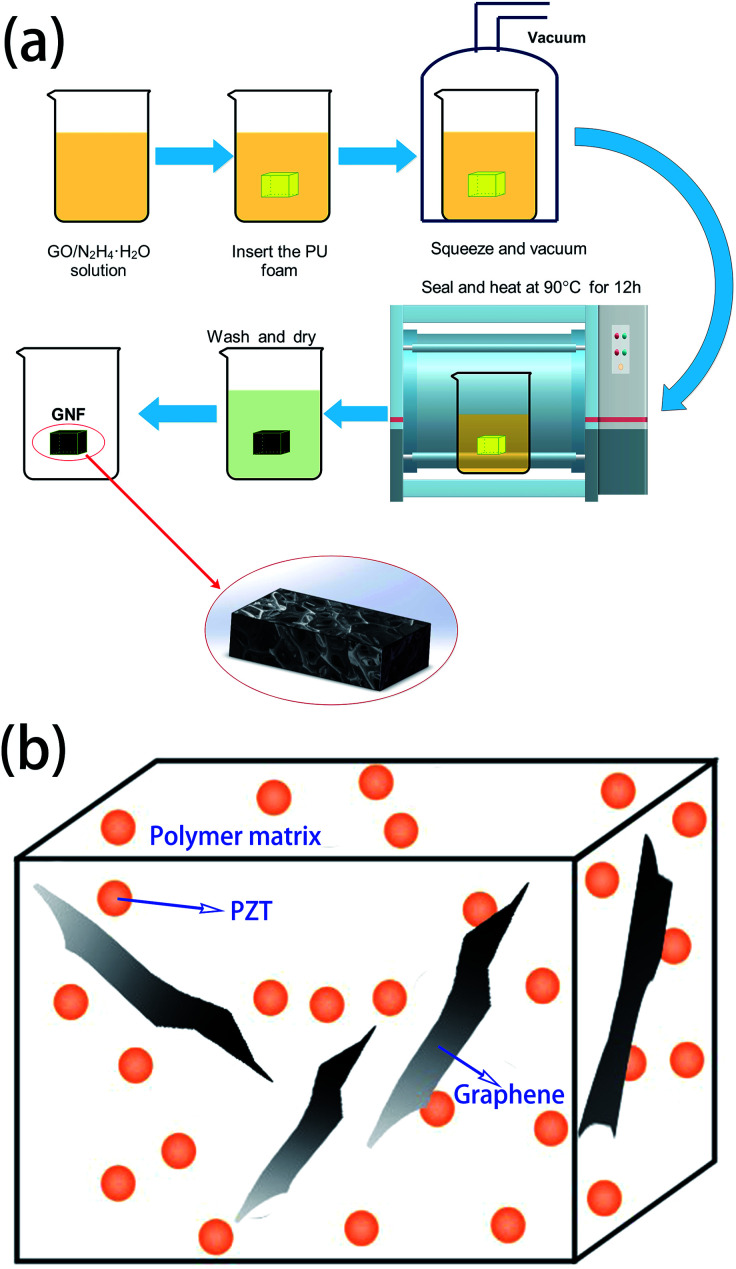
(a) Fabrication process of GNF (b) schematic structure of piezoelectric damping composite.

The synthesis process of GNF (rGO foam or 3D rGO) is shown in Fig. 1(a), showing how 3D rGO can be obtained by hydrothermal reduction of GO. The PU foam (a 20 mm × 20 mm × 5 mm cuboid) was immersed in a 50 mL beaker containing the pre-formulated solution of GO (20 mL, 0.5–7 mg mL^−1^) and hydrazine hydrate (5 μL hydrazine hydrate per mg of GO). The PU foam was then squeezed various times and placed in a vacuum oven at room temperature for 15 min to remove the bubbles trapped in the foam. Plastic film was used to seal the beaker, which was then heated at 90 °C for 12 h to reduce GO. The rGO sheets self-assembled along the PU foam backbone with π–π stacking between rGO sheets to become a 3D structure. Because rGO is insoluble in water, it has very poor affinity for glass but good affinity for PU foam. After reduction, GNF was washed with deionized water more than three times to remove impurities. The product was then dried at 70 °C for 12 h to obtain GNF. The rGO composition can be easily adjusted by varying the concentration of the GO solution.

The rGO sheets were synthesized in a similar manner. GO solution (5 mg mL^−1^) was prepared by a modified Hummers method^30^ and reduced by hydrazine hydrate (5 μL hydrazine hydrate per mg of GO). The solution was heated at 90 °C for 12 h with vigorous stirring. The compound was then frozen at −50 °C for 4 h and vacuum dried at −50 °C for 48 h to obtain the rGO sheets. The sheets were then dried at 50 °C for 12 h and set aside.

### 2.2 Fabrication of the piezoelectric damping composite

Polytetramethylene ether glycol (PTMEG, average molecular weight 2000) and 4,4′-diphenylmethane diisocyanate (MDI) were purchased from Shanghai Aladdin Biochemical Polytron Technologies Co., Ltd., China. Glycerol was supplied by Shanghai Ling Feng Reagent Co., Ltd., China. PZT (diameter ∼5 μm) was purchased from Zibo Braun Electronics Co., Ltd., China. Before use, PTMEG and glycerol were placed in a vacuum oven at 110 °C for 4 h to remove residual water and then cooled to 60 °C. MDI was also heated at 60 °C until it became a completely clear liquid. The monomers were then homogeneously mixed at a PTMEG : glycerol : MDI weight ratio of 41.96 : 7.49 : 0.55 under vigorous mechanical stirring.

PZT was added to the monomer mixture of PU with vigorously stirring at 60 °C. GNF was then immersed in the mixture, followed by squeezing more than three times and under vacuum for 15 min at room temperature to remove bubbles. The mixture was then heated at 75 °C for 4 h to allow the composite to pre-react and finally heated at 120 °C for 6 h to complete the reaction. The structure of the obtained composite is shown in Fig. 1(b), where PZT/PU is a 0–3 type composite, PZT particles are dispersed in the PU matrix, and rGO as the conductive phase intersperses among the PZT particles.

### 2.3 Fabrication of the PU/rGO sheet composite

The rGO sheets were added to the monomer mixture of PU with a of PTMEG : glycerol : MDI weight ratio of 41.96 : 7.49 : 0.55 and vigorously stirred at 60 °C for 10 min. The mixture was then heated at 75 °C for 4 h and finally heated at 120 °C for 6 h.

### 2.4 Characterization and testing

Fourier transform infrared (FTIR) spectroscopy was performed with a FTIR spectrometer (EQUINOX55, Bruker) using the KBr method in the frequency range 400–4000 cm^−1^. Raman spectroscopy was performed with a SENTERRAR200 Raman spectrometer with a 532 nm laser source. The morphology and structure were observed by field emission scanning electron microscopy (SEM, S-4800, Hitachi) with an acceleration voltage of 10 kV. The conductivity tests were performed with a four-point probe conductivity measurement device (RTS-8, Probes Tech., China) and high-resistance meter (ZC36, Shanghai Sixth Meter Co., Ltd, China). The pyrolysis processes of the samples were investigated by thermogravimetric analysis (TGA, Pyris 1, PerkinElmer) under a nitrogen atmosphere from 30 to 750 °C at a heating rate 10 °C min^−1^. The tensile strength was tested with a Zwick Z100 universal material testing machine (the samples were prepared according to ISO37:2011). Dynamic mechanical analysis was performed with a DMA8000 analyzer (PerkinElmer, USA) in compression mode at a frequency of 1 Hz from −70 to 80 °C at a heating rate of 3 °C min^−1^ (the dimensions of the samples were 8 mm × 8 mm × 1.2 mm). The piezoelectric strain constant *d*_33_ was determined with a quasi-static *d*_33_ meter (ZJ-3, Institute of Acoustics, Chinese Academy of Sciences).

## 3. Result and discussion

### 3.1 Characterization of rGO and PU

**Fig. 2:**
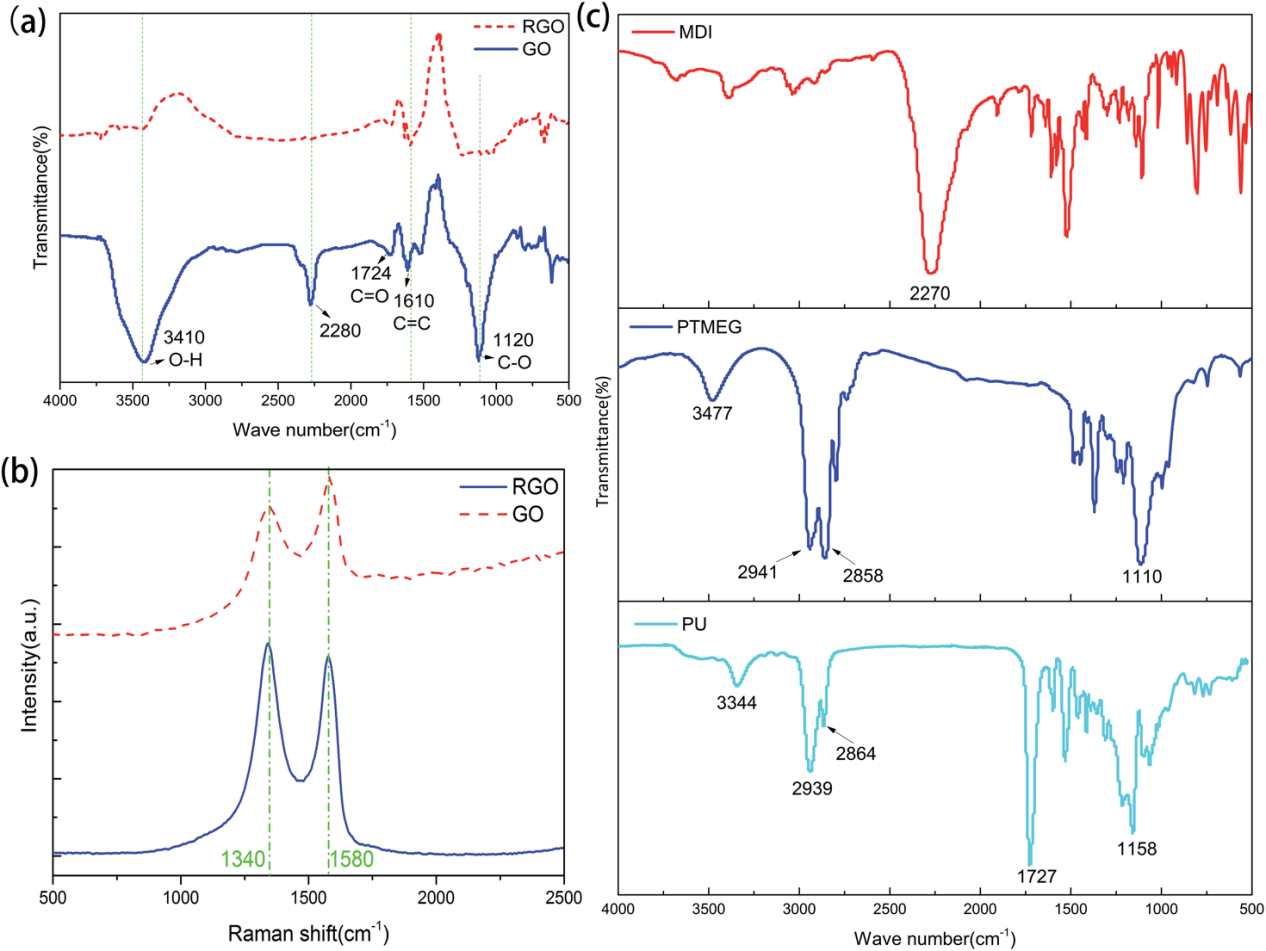
(a) FTIR spectra of GO and rGO. (b) Raman spectra of GO and rGO. (c) FTIR spectra of MDI, PTMEG, and PU.

The reduction process of GO was characterized by FTIR spectroscopy (Fig. 2(a)). For GO, the peaks at 3410 and 1724 cm^−1^ are attributed to the stretching vibrations of O–H and C=O bonds, while the peak at 1120 cm^−1^ is assigned to the presence of the C–O bond.^31^ The presence of these oxygen-containing groups suggests that graphene was successfully oxidized. For rGO, these peaks almost disappear, indicating that GO is successfully reduced. There is a strong absorption peak at 2280 cm^−1^ in the spectrum of GO, which may be caused by the ammonium salts generating during oxidation of graphite. This peak almost disappears after reduction of GO, indicating that these impurities are removed without affecting the structure of rGO. Raman spectroscopy was performed to verify conversion of GO in the reduction process. As shown in Fig. 2(b), there are two broad peaks at 1340 and 1580 cm^−1^, which can be ascribed to the D band (associated with the extent of structural disorder) and G band (related to sp^2^ hybridization).^32^ The ratio of the peak intensities of the D band to the G band of GO (0.84) increases when GO is reduced to rGO (1.07) because the reduction process removes oxygen-containing groups and results in a relatively high defect ratio. It also confirms that GO is successfully reduced.

Because the PU monomers could not be stirred after incorporating with GNF, FTIR spectroscopy was performed to verify whether the reaction conditions are appropriate. The PU monomers were directly mixed and polymerized without stirring. As shown in Fig. 2, several peaks appear in the spectrum of PU after the reaction. The peaks at 1727 and 1158 cm^−1^ can be ascribed to the C=O and C–O bonds of –NHCOO^−^, respectively, while the peak at 3344 cm^−1^ can be assigned to the stretching vibration of N–H without hydrogen bonding, suggesting synthesis of PU. The peaks of the reactive functional groups of PTMEG and MDI (the characteristic peak of the isocyanate group at 2270 cm^−1^ and the peak of the association state hydroxyl group at 3477 cm^−1^) are not present, indicating that there are no residual hydroxyl and isocyanate groups and PTMEG and MDI completely reacted. Moreover, there are no peaks between 1640 and 1690 cm^−1^ corresponding to the C=O stretching vibration of the urea group, which indicates that no urea groups formed. Therefore, it can be concluded that the reaction conditions are suitable and the resulting PU does not contain unreacted hydroxyl and isocyanate groups generated at relatively low reaction temperature or urea groups generated at very high temperature.

### 3.2 Performance of the PU/PZT composites

**Fig. 3:**
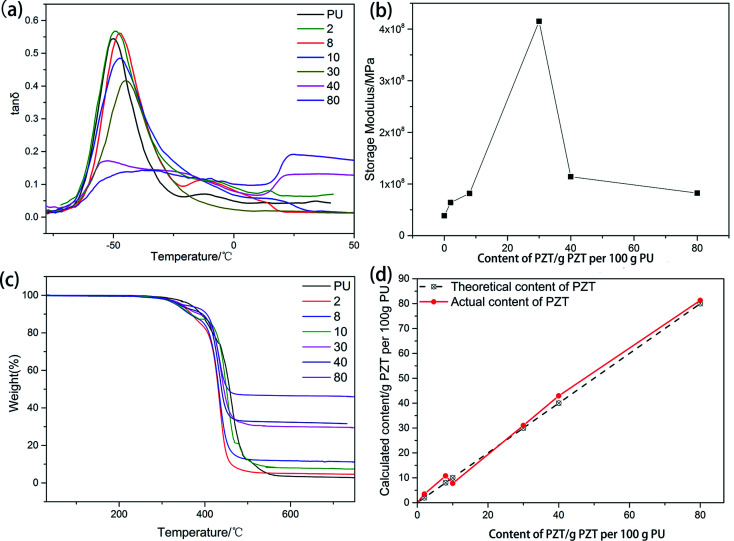
(a) Loss factor, (b) storage modulus at −70 °C, (c) TGA, and (d) actual PZT content calculated from TGA of composites with 0, 2, 8, 10, 30, 40, and 80 g PZT per 100 g PU.

**Fig. 4:**
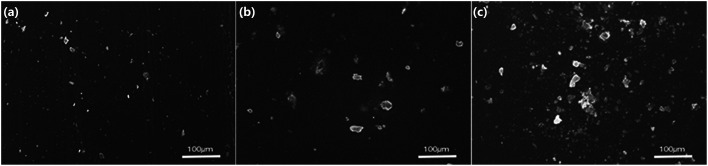
SEM images of composites with (a) 2, (b) 8, and (c) 24 g PZT per 100 g PU.

Fig. 3(a) shows the dynamic properties of the composite consisting of PU and PZT without rGO. The glass transition temperature (*T*_g_) increases with increasing PZT content because the PZT particles hinder the motion of the molecular chain segments, which can also be seen from Table 1. Moreover, with increasing PZT content, the damping properties (characterized by the loss factor tan *δ* at *T*_g_ and the temperature range where tan *δ* ≥ 0.3) of the composites first increase and then decrease. The maximum tan *δ* is achieved when the PZT content is 2 g per 100 g PU. Thus, the composite material with 2 g PZT per 100 g PU was chosen as the matrix of the piezoelectric damping composite. Addition of PZT has two opposite effects on the damping properties of the composite. On the one hand, PZT dilutes the matrix, which decreases the damping properties. On the other hand, addition of PZT introduces friction losses of the PZT particles and PZT with the PU matrix to increase mechanical energy dissipation. At a low PZT content, the second effect is dominant, but the first effect is dominant when the PZT content is high. This is the reason why there is an optimal PZT content to achieve the best damping performance. Furthermore, when the PZT content is more than 40 g per 100 g PU, crosslinking of molecular chains of PU is blocked, so tan *δ* dramatically decreases (tan *δ* < 0.3 in the temperature range from −75 to 50 °C) and then increases at about 20 °C. Similar behavior is observed for the relationship between the storage modulus and the PZT content (Fig. 3(b)). The difference is that the storage modulus reaches a maximum of 420 MPa at a higher PZT content of about 30 g PZT per 100 g PU. According to the SEM images of the specimens with 2, 8, and 24 g PZT per 100 g PU (Fig. 4), dispersion of PZT gradually decreases as the PZT content increases. The lack of dispersion is probably the reason why the storage modulus decreases as the PZT content increases. However, the damping properties and elastic modulus dependency on the PZT content are attributed to different physical principles. The elastic modulus should be proportional to the content of PZT in a well-distributed situation, so the elastic modulus begins to decrease at a higher PZT content than tan *δ*.

**Table 1:** Effect of the PZT content on the damping properties

Sample	*T*_g_ (°C)	Loss factor (tan *δ*) at *T*_g_	Temperature range (°C) where tan *δ* ≥ 0.3
PU	−49.8	0.54	15.8 (−56.9 to −41.1 °C)
2	−49.5	0.57	20.3 (−57.6 to −37.3 °C)
8	−47.5	0.56	17.6 (−54.6 to −37.0 °C)
10	−47.5	0.48	18.5 (−55.2 to −36.7 °C)
30	−44.8	0.42	13.8 (−51.2 to −37.4 °C)

TGA of PU with different PZT contents is shown in Fig. 3(c). The thermal stability of PU decreases with increasing PZT content (the temperatures of 5% thermal weight loss are given in Table 2). There are two factors that can influence the stability of the composite. First, there are some hydroxyl groups on the surface of PZT that can react with isocyanate groups to create crosslinking points of the composite, which is good for the stability. Second, the hydroxyl groups have a negative effect on the synthesis of PU, which has a greater effect on the thermal stability of the composite and decreases the thermal stability.

**Table 2:** Effect of the PZT content on the thermal stability

Sample	PU	2	8	10	30	40	80
Temperature of 5% thermal weight loss (°C)	366	343	334	336	347	339	348

Using the method of *in situ* polymerization of PU followed by addition of PZT, PZT may be unevenly distributed and biased in the direction of gravity. From the TGA curve of the composite, the actual PZT content can be calculated by1
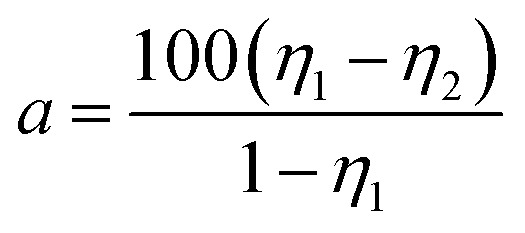
where *a* is the actual PZT content per 100 g PU, *η*_1_ is the TGA residual weight of the composite at 700 °C, and *η*_2_ is the TGA residual weight of pure PU at 700 °C. The calculated results are shown in Fig. 3(d) (solid line). Although the actual and theoretical contents are not identical, the error is less than 6% and acceptable.

### 3.3 Performance of the piezoelectric damping composites

**Fig. 5:**
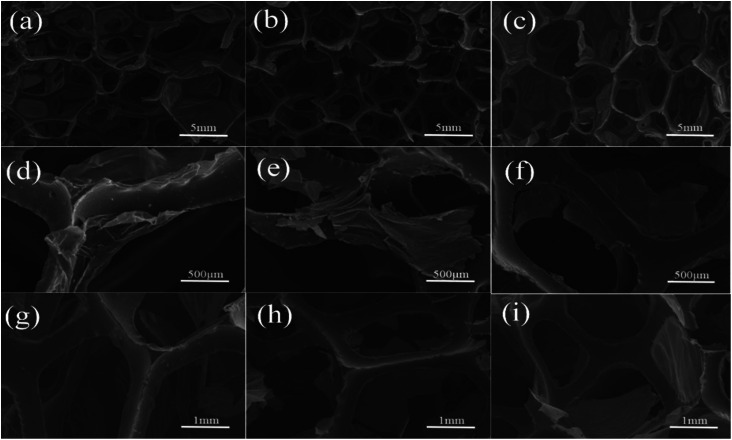
SEM images of GNF with calculated rGO contents of (a) and (d) 2.6 wt%, (b) and (e) 10.2 wt%, (c) and (f) 18.0 wt%, (g) 25.4 wt%, (h) 30.0 wt%, and (i) 44.7 wt%.

**Fig. 6:**
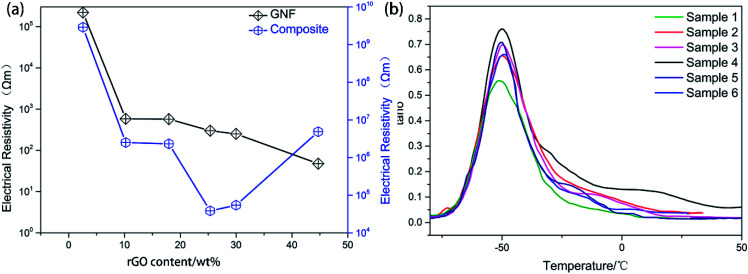
(a) Relationship between the electrical resistivity and the rGO content for GNF and the composite. (b) Dynamic properties of composites with GNF of 2.6 wt% (sample 1), 10.2 wt% (sample 2), 18.0 wt% (sample 3), 25.4 wt% (sample 4), 30.0 wt% (sample 5), and 44.7 wt% (sample 6).

GNF was characterized by SEM at different resolutions to clearly visualize the morphological distribution of rGO (Fig. 5). The conductivity was measured by a four-point probe conductivity measurement device and high resistance meter (Fig. 6(a)). The rGO percentage weight of GNF was determined by (*W*_2_ − *W*_1_)/*W*_2_ × 100%, where *W*_1_ is the weight of the PU skeleton after heating at 70 °C for 1 h and *W*_2_ is the sample weight after synthesis of GNF. From Fig. 5(a)–(c), GNF is formed by rGO adhering to the PU skeleton, and the morphology of GNF is the same as that of the skeleton. As the rGO content increases, its morphological distribution and the electrical conductivity significantly change. The conductivity of GNF continuously increases whereas the conductivity of the composite initially increases and then decreases. For rGO content of 2.6 wt%, connection of rGO is incomplete and the resistance is higher than 2.2 × 10^5^ Ω m. The corresponding composite is almost an insulator with a resistance of about 9 × 10^9^ Ω m. For rGO content of 10.2 wt%, the rGO sheets start to overlap on the skeleton and the conductivity drastically decreases by almost three orders of magnitude. When the rGO content is 18 wt%, the rGO sheets attached to the PU skeleton extensively overlap. When the rGO content is 44.7 wt%, rGO begins to fill the skeleton's voids. Surprisingly, even though the conductivity of GNF increases, the conductivity of the composite decreases. This behavior can be attributed to the fact that the rGO sheets are severely damaged because of the very high overlap rate when the mixture of PU and PZT is injected into the composite. From the comparison of the conductivity, there is a difference of at least three orders of magnitude between the conductivities of GNF and the composite. This is because introduction of PU and PZT severely decreases the electrical conductivity and disrupts the original conductive structure of GNF. The conductivity percolation threshold of the composite is reached for GNF with 25.4 wt% rGO, which has a resistance of about 4.8 × 10^4^ Ω m.

**Fig. 7:**
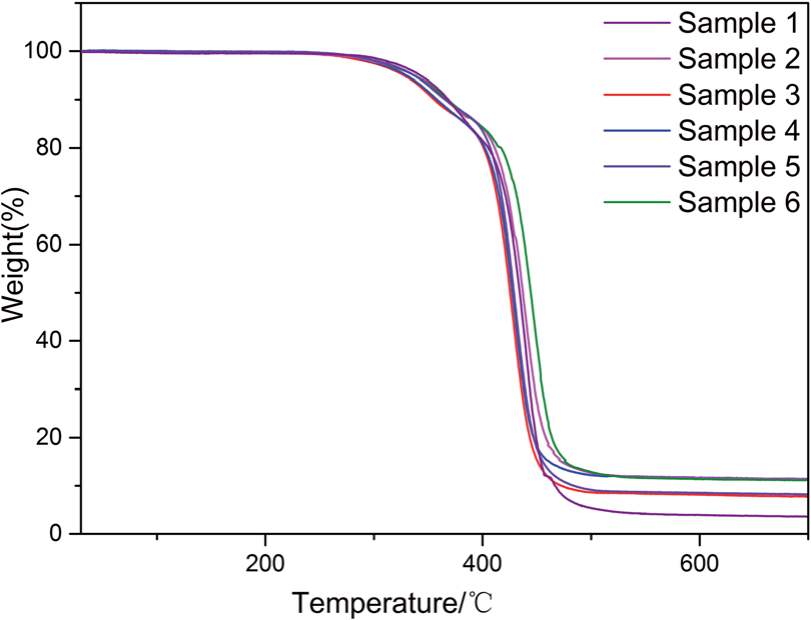
TGA of composites with 2.6 (sample 1), 10.2 (sample 2), 18.0 (sample 3), 25.4 (sample 4), 30.0 (sample 5), and 44.7 wt% (sample 6) rGO content.

Through introduction of piezoelectric and conductive phases, the vibration energy can be dissipated in five ways: (1) friction between polymer segments, (2) friction between polymer chains and PZT, (3) friction between PZT particles, (4) polarization loss of piezoelectric ceramics under alternating stress, and (5) dissipation by the way of vibration energy, electric energy, and heat energy. It follows that the piezoelectric damping composites show a wider temperature range and better loss factor than ordinary composites that can only use the first three dissipation modes. From Tables 1 and 3, all of the piezoelectric damping composites have better damping performance than the PU matrix and PU/PZT composites. The dynamic properties and TGA curves of composites with 2 g PZT per 100 g PU and different rGO contents are shown in Fig. 6(d) and 7. The tan *δ* value at *T*_g_ initially increases and then decreases with increasing rGO content, and it is positively related to the conductivity of the composite. Furthermore, the best damping performance of the composite is achieved with rGO content of 25.4 wt% (sample 4). In this case, the rGO content is 0.70 wt% in the whole composite and tan *δ* = 0.76 at −50.2 °C (Table 3). The loss factor is 0.22 (about 41%) higher and the temperature range where tan *δ* ≥ 0.3 is 13.2 °C (almost 84%) wider than those of the PU matrix. Moreover, the tan *δ* value at *T*_g_ is also higher than that of the composite without rGO (usually 0.02–0.03). In addition, the thermal stabilities of the samples are close to that of the temperatures of 5% thermal weight loss (Table 4, between 328 (sample 3) and 342 °C (sample 1)), so introduction of rGO does not significantly affect the thermal stability.

**Table 3:** Influence of the rGO content on the damping behavior

Sample	*T*_g_ (°C)	Loss factor (tan *δ*) at *T*_g_	Temperature range (°C) when tan *δ* ≥ 0.3
1	−51.0	0.56	22.5 (−60.4 to −37.9 °C)
2	−49.5	0.65	27.9 (−60.7 to −33.8 °C)
3	−50.0	0.70	25.3 (−60.2 to −35.9 °C)
4	−50.2	0.76	29.0 (−61.5 to −32.5 °C)
5	−50.6	0.71	22.2 (−59.9 to −37.7 °C)
6	−49.7	0.66	22.4 (−60.0 to −37.6 °C)

**Table 4:** Influence of the rGO content on the thermal stability

Sample	1	2	3	4	5	6
Temperature of 5% thermal weight loss (°C)	342	340	328	332	340	337

**Fig. 8:**
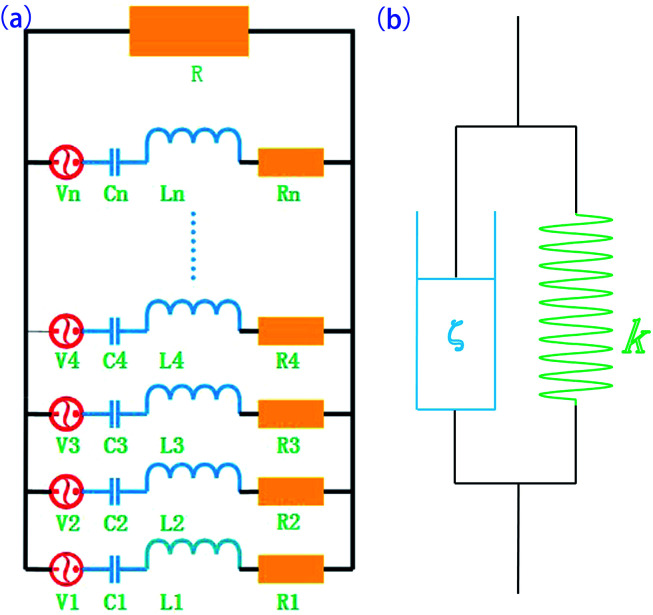
Models of the (a) equivalent electrical circuit and (b) equivalent spring–dashpot model.

For the five dissipation paths mentioned above, friction of the polymer and the piezoelectric effect are responsible for the largest dissipation. The equivalent model of the piezoelectric damping composite is shown in Fig. 8. When alternating stress is applied, the energy can be dissipated by the two following ways: (1) the viscoelastic behavior of the polymer matrix, which can described by an equivalent dash-pot and spring in parallel, where the dash-pots dissipate the mechanical energy while the springs store the strain energy. (2) The piezoelectric material converts the mechanical energy into electrical energy, which is stored on the surface of piezoelectric material. If the equivalent circuit resistance is suitable, some of the accumulated charge in the PZT can be dissipated. Piezoelectric materials can be represented by the combination of the power source, resistance, capacitance, and inductance.

According to the theory of Law *et al.*,^33^ the overall optimal resistance for the maximum energy dissipation in the circuit *R* can be approximated by2
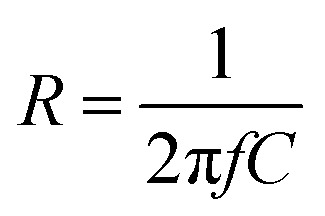
where *C* is the capacitance of the piezoelectric material in the poling direction at low frequency in the free-stress state and *f* is the frequency of the applied force. From eqn (1), there is an optimal value of *R* for a specific frequency. Because the resistivity of the material does not linearly change with the content of the conductive phase, the optimal resistance value often occurs at the percolation threshold, at which a sudden change of the conductivity will occur.

**Fig. 9:**
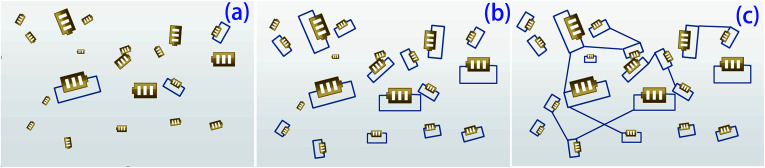
Models of composites with different rGO contents. (a) Low graphene content, (b) proper graphene content, and (c) high graphene content.

**Fig. 10:**
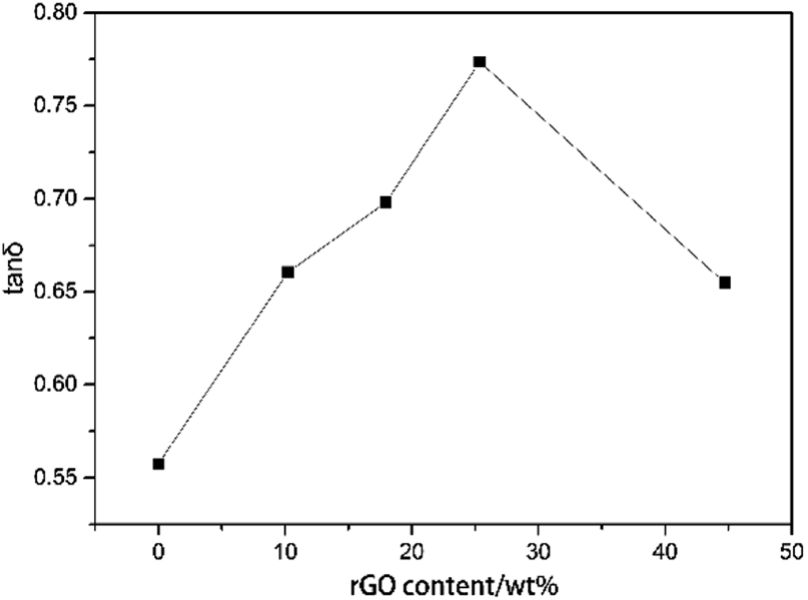
Relationship between tan *δ* at *T*_g_ and the rGO content.

Schematic representations of the composites with different rGO contents are shown in Fig. 9. The composites with 10.2, 25.4, and 44.7 wt% rGO are represented by Fig. 9(a)–(c), respectively. The amount of the conductive phase in Fig. 9(a) is insufficient for the electricity generated by the PZT particles to be significantly dissipated. In this case, the composite produces an internal polarization electric field that eventually transforms into mechanical energy. In Fig. 9(c), the PZT particles are connected because of the high rGO content. The PZT particles charge and discharge each other, so energy consumption in the system is relatively small. For the case represented by Fig. 9(b), the conductive phase content is such that the PZT particles act as separate shunt power sources, so this scenario is ideal to maximize the loss factor and for mechanical energy–electricity energy–heat energy transformation. This scenario corresponds to the *R* value calculated by eqn (2). From Fig. 10, sample 4 has the highest tan *δ* at *T*_g_. Furthermore, the *d*_33_ value of sample 1 is about 8 pC N^−1^. This means that introduction of the piezoelectric material has produced a piezoelectric effect.

**Fig. 11:**
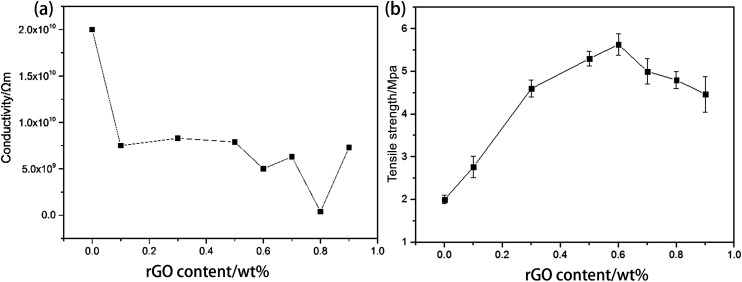
(a) Conductivity and (b) tensile strength of the PU/rGO composites.

Too much conductive phase can sometimes affect the mechanical properties of the matrix. As a control experiment with all of the other conditions kept the same, the tensile strength and conductivity of composites with various contents of rGO sheets without 3D structure are shown in Fig. 11. From Fig. 11(b), the tensile strength significantly increases with increasing rGO content. The highest tensile strength is about 5.5 MPa with 0.6 wt% rGO, which is almost 2.5 times higher than that of pure PU. For rGO > 0.6 wt%, the tensile strength decreases with increasing rGO content. Thus, it is advisable that the rGO content should be not much higher than the optimal value of 0.6 wt%. However, the lowest conductivity of about 3.5 × 10^9^ Ω m is obtained with rGO content of 0.8 wt% (Fig. 11(b)), and this can still be considered to be an insulator material. When the rGO content is very high, PU has almost no polymerization, and the rGO content cannot be further increased by the *in situ* polymerization method. It can be concluded that the conductive threshold is more than 0.9 wt%. Thus, the 3D rGO structure can significantly reduce the use of the conductive phase (about 0.7 wt%), and it has a small effect on the tensile strength of the matrix.

## 4. Conclusion

GNF and PU have been successful fabricated by the hydrothermal reduction method and *in situ* polymerization, respectively. The morphology of GNF is similar to that of the PU foam skeleton because rGO self-assembles on the PU framework. The conductivity of GNF is highest at 44.7 wt% rGO, but the best conductivity is achieved for GNF with 25.4 wt% rGO. TGA shows that introduction of PZT decreases the thermal stability of PU and rGO has little effect on the thermal stability. Through other dissipative channels, including polarization loss of piezoelectric ceramics and vibration–electric-heat dissipation, the piezoelectric damping composites cover a wider temperature range and have better damping properties than the PU matrix. The damping performance is the best when the rGO content is 0.70 wt% of the whole composite, resulting in tan *δ* = 0.76 at −50.2 °C. The loss factor is 0.22 (almost 41%) higher and the temperature range where tan *δ* ≥ 0.3 is 13.2 °C (almost 84%) wider than those of the PU matrix. Through a controlled experiment, there is a significant decrease of conducive phase usage (from more than 0.9 wt% to 0.7 wt%) by introduction of the 3D rGO structure, and this does not significantly decrease the tensile strength.

## Conflicts of interest

There are no conflicts to declare.

## Supplementary Material

RA-008-C8RA00175H-s001
