# Supplementary material for: High loss factor piezoelectric damping composite with three-dimensional reduced graphene oxide as the conductive phase
Source: RSC Adv. 2018 Apr 3;8(23):12494–502. doi: 10.1039/c8ra00175h (PMC9079627; doi:10.1039/c8ra00175h)
Supplement: RA-008-C8RA00175H-s001 [file RA-008-C8RA00175H-s001.pdf]

Electronic Supplementary Material (ESI) for RSC Advances.
This journal is © The Royal Society of Chemistry 2018

Supporting information

Materials: Natural graphite powder (40 μm with purity >99.85%) was supplied by Xiamen Knano Graphene Technology Co., Ltd, and hydrazine hydrate (85%), H_2SO_4, $NaNO_3$, $KMnO_4$, H_2O_2 were purchased from Sinapharm Chemical Reagent Co., Ltd, China.

Preparation of graphene oxide (GO): In a typical experiment, graphite powder (3 g) was placed in a 1000 mL beaker, and then 80 mL H_2SO_4 solution and 1.5 g $NaNO_3$ were added in an ice-water bath with stirring. The mixture was kept at 0 °C for 40 min and then 9 g $KMnO_4$ was gradually added and the mixture was heated at 35 °C for 1 h. The mixture was then diluted with 200 mL of 30 °C deionized (DI) water and heated at 60 °C for 1 h until the color of the solution became dark brown. H_2O_2 (10 mL) was then added to the mixture under stirring and the color changed to brilliant yellow along with bubbling. Centrifugation at relative centrifugal force of 9900 × g for 10 min was then performed to separate GO and the liquid. The obtained solid was washed with 1:10 HCl aqueous solution (1 L) to remove metal ions, centrifuged (7000 × g for 5 min), washed with 1 L DI water, and centrifuged (7000 × g for 5 min) more than 3 times to remove impurities. The product was dissolved in 500 mL DI water and ultrasonicated for 6 h. The solution was centrifuged (4000 × g for 5 min) and the obtained liquid was GO solution.

The GO obtained was multilayered and had a size of 2-20 μm (a small amount of single layer GO also could be found shown in Fig. S1). The concertation GO was determined by $W_1/W_2 \times 100\%$, where W_2 and W_1 are the weight of the 25mL GO solution before and after heating at 50 °C for 12 h respectively.

The size of rGO sheets (the GO that is reduced by hydrazine hydrate) ranges from 10-200 μm shown in Fig. S2.

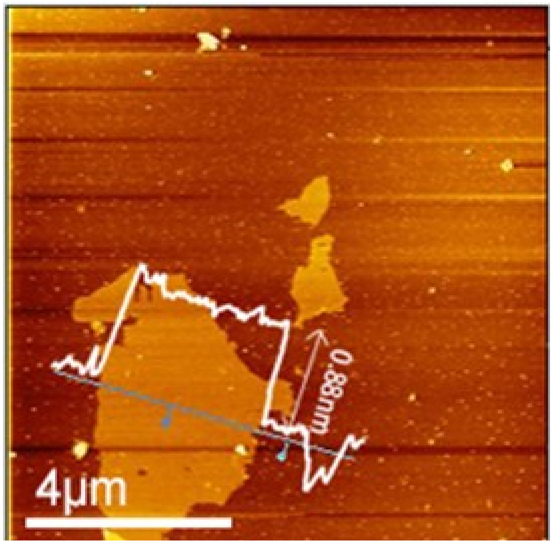

Fig. S1 The atomic force microscope (Nanonavi E-Sweep, SEIKO) of single-layer GO sheet

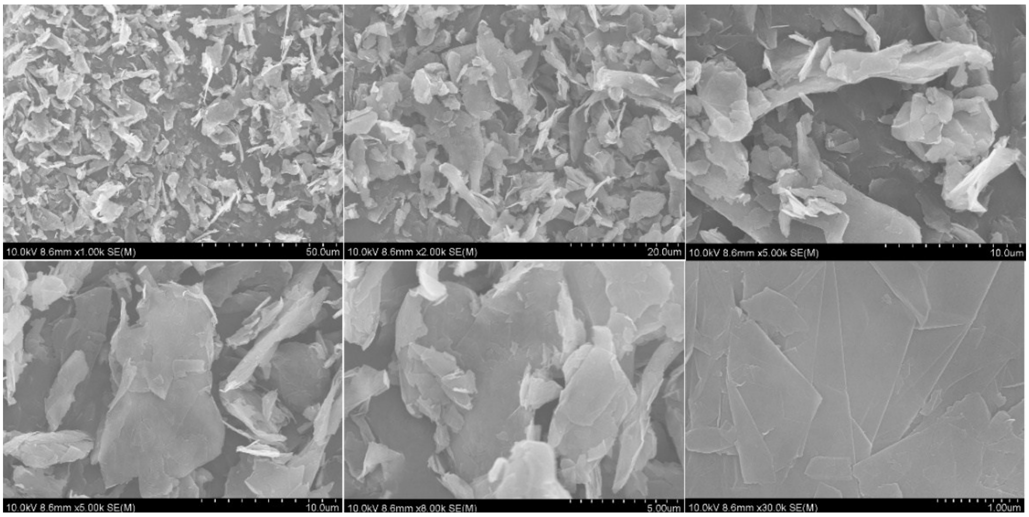

Fig. S2 SEM images of rGO sheets
